# Differential and coordinated expression of defensins and cytokines by gingival epithelial cells and dendritic cells in response to oral bacteria

**DOI:** 10.1186/1471-2172-11-37

**Published:** 2010-07-09

**Authors:** Lei Yin, Takahiro Chino, Orapin V Horst, Beth M Hacker, Edward A Clark, Beverly A Dale, Whasun O Chung

**Affiliations:** 1 Department of Oral Biology, School of Dentistry, University of Washington, 1959 NE Pacific Street, Seattle, WA, 98195, Box 357132, USA; 2 Department of Dermatology, School of Medicine, University of Washington, 1959 NE Pacific Street, Seattle, WA, 98195, Box 357132, USA; 3 Department of Immunology, School of Medicine, University of Washington, 1959 NE Pacific Street, Seattle, WA, 98195, Box 357242, USA; 4 Department of Microbiology, School of Medicine, University of Washington, 1959 NE Pacific Street, Seattle, WA, 98195, Box 357242, USA

## Abstract

**Background:**

Epithelial cells and dendritic cells (DCs) both initiate and contribute to innate immune responses to bacteria. However, much less is known about the coordinated regulation of innate immune responses between GECs and immune cells, particularly DCs in the oral cavity. The present study was conducted to investigate whether their responses are coordinated and are bacteria-specific in the oral cavity.

**Results:**

The β-defensin antimicrobial peptides hBD1, hBD2 and hBD3 were expressed by immature DCs as well as gingival epithelial cells (GECs). HBD1, hBD2 and hBD3 are upregulated in DCs while hBD2 and hBD3 are upregulated in GECs in response to bacterial stimulation. Responses of both cell types were bacteria-specific, as demonstrated by distinctive profiles of hBDs mRNA expression and secreted cytokines and chemokines in response to cell wall preparations of various bacteria of different pathogenicity: *Fusobacterium nucleatum*, *Actinomyces naeslundii *and *Porphyromonas gingivalis*. The regulation of expression of hBD2, IL-8, CXCL2/GROβ and CCL-20/MIP3α by GECs was greatly enhanced by conditioned medium from bacterially activated DCs. This enhancement was primarily mediated via IL-1β, since induction was largely attenuated by IL-1 receptor antagonist. In addition, the defensins influence DCs by eliciting differential cytokine and chemokine secretion. HBD2 significantly induced IL-6, while hBD3 induced MCP-1 to approximately the same extent as LPS, suggesting a unique role in immune responses.

**Conclusions:**

The results suggest that cytokines, chemokines and β-defensins are involved in interaction of these two cell types, and the responses are bacteria-specific. Differential and coordinated regulation between GECs and DCs may be important in regulation of innate immune homeostasis and response to pathogens in the oral cavity.

## Background

Epithelia throughout the body function as a physical barrier against invading bacteria and also provide effective innate immune defenses by producing antimicrobial peptides [1-4]. The β-defensins are antimicrobial peptides that are widely expressed in epithelial tissues including the oral cavity [5-7]. They have a broad spectrum of activity against both Gram-negative and Gram-positive bacteria as well as some fungi and viruses [2,8]. In addition to their direct antimicrobial activity, human β-defensins (hBDs) also directly stimulate antigen-presenting dendritic cells (DCs) and memory T cells, and thus can link innate and adaptive immune responses [9-11]. They also provoke efficient epithelial barrier repair to limit entry of invading bacteria [12]. Antimicrobial peptides provide multiple benefits as frontline defense molecules, and are particularly important in the oral cavity in which the health of the tissue depends on the balance between commensal (non-pathogenic) and pathogenic microbes and host defenses.

Human β-defensin 1 (hBD1) is constitutively expressed by gingival epithelial cells (GECs), while the expression of hBD2 and hBD3 is more variable and inducible. Epithelial cells produce hBD2 following stimulation with microorganisms (Gram-negative, Gram-positive bacteria and *Candida albicans*) or cytokines such as TNF-α and IL-1β[13-15]. The expression of the inducible hBD2 in GECs *in vitro *is regulated by several distinct signaling pathways, depending on the oral bacterial species. Commensal bacteria such as *Fusobacterium nucleatum *and *Streptococcus gordonii *induce hBD2 via MAPK pathways, while periodontal pathogens such as *Porphyromonas gingivalis *and *Aggregatibacter actinomycetemcomitans *also signal via NF-κB; in addition, *P. gingivalis *signals via protease-activated receptors [16-19]. This suggests that GECs can distinguish commensal from pathogenic bacteria. Nevertheless, purified bacterial LPS is a poor stimulant for hBD2, and *in vitro *studies show that hBD2 induction is greatly amplified in epithelial cells when monocyte/macrophage-like cells are included in the culture system [20,21].

Intestinal epithelial cells cross-talk with DCs, and coordinately regulate the gut homeostasis in response to different bacteria [22]. DCs in the lamina propria take up bacteria directly in the gut, [23,24] and mucosal DCs induce divergent cytokine responses in response to commensal and pathogenic bacteria [25]. In oral mucosa in response to bacteria, GECs and DCs produce a wide range of cytokines and chemokines, and DCs may play a critical role during immune/inflammatory responses to specific components within biofilms as part of the pathogenesis of periodontal disease [26-28]. While there is considerable information about innate responses in epidermal keratinocytes and pulmonary epithelial cells [20,21], much less is known about the coordinated regulation of innate immune responses between GECs and immune cells, particularly DCs in the oral cavity. We hypothesized that DCs and GECs coordinately regulate specific innate immune responses in response to oral bacteria. We used primary GECs and monocyte-derived DCs to examine differential responses between these two cell types *in vitro*. Here we report that expression of β-defensins by both cell types is dependent on the bacterial species, and that IL-1β from DCs mediates expression of multiple responses in GECs. In addition, GECs also affect the immune responses of DCs via their expression of defensins. Our results underscore the importance of functional coordination between GECs and DCs for promoting characteristic innate immune responses in the oral cavity.

## Methods

### Chemicals and Reagents

Bacterial crude cell wall extracts from *F. nucleatum *(ATCC 25586), *Actinomyces naeslundii *(ATCC19039), and *P. gingivalis *(ATCC 33277) (FnCW, AnCW and PgCW, respectively) were prepared as previously described [14,29]. Antibodies used include mouse IgG anti-human Langerin (Novocastra Laboratory Ltd., Newcastle, UK), rabbit polyclonal anti-hBD1 serum (kindly provided by Dr. Tomas Ganz, UCLA), rabbit polyclonal anti-hBD2 (Alpha Diagnostic International, San Antonio, TX) and secondary antibodies biotin-conjugated donkey anti-mouse (Jackson ImmunoResearch, West Grove, PA), FITC-conjugated goat anti-rabbit (Vector Laboratories, Burlingame, CA), FITC-conjugated donkey anti-goat IgG (Jackson ImmunoResearch), and Texas Red-conjugated streptavidin (Vector Laboratories). Recombinant human IL-1 receptor antagonist (IL-1ra) was purchased from R&D Systems (Minneapolis, MN). Human β-defensin 2 and 3 were purchased from Peptides International (Louisville, KY).

### Human primary gingival epithelial cell culture, stimulation and oral tissue model

Healthy human gingival tissue samples were obtained from patients undergoing third-molar extraction at the Oral Surgery Clinic, School of Dentistry, University of Washington in accordance with IRB-approved procedures. Cells were prepared for culture as previously described [29]. The isolated primary human GECs were cultured in serum-free keratinocyte basal medium supplemented with keratinocyte growth factors (Cambrex, Walkersville, MD) and grown to 80% confluence before treatment with different stimulants. In some experiments, GECs were treated before stimulation with human IL-1 receptor antagonist, IL-1ra (100, 200, 400 ng/ml).

The organotypic tissue model consisted of normal human oral keratinocytes, fibroblasts, and DCs (containing plasmacytoid DC and myeloid DC) in serum-free medium to form a three-dimensional differentiated full thickness tissue, which histologically is similar to gingival mucosa (ORL-100, EpiOralFT™, MatTek Corporation, Ashland, MA). Upon receipt, individual cell culture inserts were placed at the air liquid interface in 6-well plates with 5 ml of serum-free minimal media containing growth factors (MatTek proprietary media) and rested overnight (37°C, 5% CO_2_). Cultures were treated topically with hBD2 or controls for 24 h, then fixed in 10% formalin, embedded in paraffin, and sectioned for immunostaining.

### Generation of monocyte-derived immature DC and treatments

Immature DCs (iDCs) were generated from human peripheral blood mononuclear cell (PBMC), obtained from healthy donors in accordance with approved IRB procedures by Ficoll-Hypaque centrifugation, and negative and positive selection [30]. To obtain iDC, CD14^+ ^monocytes were seeded at a density of 1 × 10^6 ^cells in 12-well plates in 2 ml RPMI-1640 media supplemented with 10% fetal bovine serum (BioWhittaker, Walkersville, MD), human granulocyte-macrophage colony-stimulating factor (100 ng/ml, Leukine; Amgen, Seattle, WA), and human IL-4 (30 ng/ml; RDI, Flanders, NJ) and cultured for 6 days. Cells were fed on days 2 and 4 by replacing half the medium and adding fresh cytokines. On day 6, the cells exhibited an iDC phenotype, (CD1a^high ^CD14^-^). For maturation of the iDCs, cells were cultured for a further 24 h in the presence of *E. coli *lipopolysaccharide (LPS; 1 μg/ml; Sigma-Aldrich Inc., St Louis, MO), or with graded doses of oral bacterial cell wall extracts (0.01 to 10 μg/ml). The maturation of iDCs into mature DCs (mDCs) was confirmed by FACS analysis for cell surface expression of CD83 as described previously [30]. Conditioned media were collected from iDCs treated with FnCW (10 μg/ml) or PgCW (10 μg/ml) for 48 h. The resulting conditioned media (CM-DCs) were briefly centrifuged and diluted (1:2; 1:20) for stimulation of GECs.

### RNA isolation and quantitative Real-time PCR

Total RNA was extracted using TRIzol (Invitrogen, Carlsbad, CA) according to the manufacturer's suggestions. Reverse transcription was performed with 2 μg of total RNA (Ambion Inc., Austin, TX). Controls without reverse transcriptase were included in each experiment. Amplification of cDNA was carried out under standard condition. Ribosomal phosphoprotein (RPO) was used as a housekeeping gene. Quantitative real-time PCR was conducted using the iCycler system (Bio-Rad, Hercules, CA) with Brilliant SYBR Green QPCR Master Mix (Stratagene, La Jolla, CA). Each reaction contained 12.5 μl of SYBR Green mix, 2 μl of cDNA, and 2 μM primers. The amplification conditions were initial denaturation at 95°C for 12 min followed by 40 cycles of denaturation at 95°C for 30 s, annealing at 57-65°C for 30 s, and elongation at 72°C for 60 s. Melt-curve analysis was performed to confirm that the signal was that of the expected amplification product. In initial experiments, amplification efficiency was determined for all primer pairs. The primer pairs used for quantitative real-time PCR were as follows: hBD1: forward 5'-CACTTGGCCTTCCCTCTGTA, reverse 5'- CGCCATGAGAACTTCCTACC; hBD3: forward 5'- GTGAAGCCTAGCAGCTATGAGGAT, reverse 5'- TGATTCCTCCATGACCTGGAA. The oligonucleotides for RPO, hBD2, IL-8, CCL20 and CXCL2 (GROβ) have been described previously [14,31]. Real-time PCR was performed in duplicate and normalized to housekeeping gene RPO. Results are expressed as the relative fold increase of the stimulated over the controls, referred to as Pfaffl's method [32].

HBD1, hBD2, hBD3 and RPO were amplified with specific primers and the PCR products were cloned with the TOPO TA Cloning kit (Invitrogen). The pCR 2.1-TOPO vector with the gene inserted was purified with PureLink Quick Plasmid Miniprep Kit (Invitrogen) and linearized with EcoRI (Promega, Madison, WI) restriction digest. The linearized plasmid was visualized and quantified by electrophoresis on a 1% agarose gel stained by ethidium bromide. Each Real-time PCR was run with duplicates of a series of seven ten-fold dilutions of the standard plasmid and a no-template control. The standard curve in each run was constructed by plotting seven dilutions of the standard plasmid DNA against the corresponding threshold cycle value. The expression of hBD1, hBD2 and hBD3 in GECs and DCs was calculated by the standard curves of the samples containing known amounts of plasmid versus absolute expression value of RPO.

### Detection of cytokines in culture supernatants

Human Cytokine Arrays III and V (Ray Biotech Inc., Norcross, GA) were prepared as described by the manufacturer and used as a screening tool for detection of multiple cytokines in culture media collected from unstimulated and bacteria-stimulated GECs and DCs.

After iDCs were treated with various stimulants, the supernatants were harvested, and levels of IL-1β, IL-6 and MCP-1 in culture medium were quantified by a sandwich ELISA technique (eBioscience, San Diego, CA). Samples were analyzed in duplicate following the manufacturer's protocol. The detection limit of ELISA was 4 pg/ml.

### Detection of β-defensin-2 and β-defensin-3 in GECs

GECs were grown in six-well plates and were treated at 80% confluence with AnCW (10 μg/ml), FnCW (10 μg/ml), PgCW (1 μg/ml) or LPS (1 μg/ml) for 24 and 48 h. Cells were washed three times with ice-cold PBS and incubated with cell lysis buffer (Cell Signaling, Danvers, MA) for 5 min at 4°. Protein extracts were obtained after sonication of cell lysates and centrifugation at 7500 g at 4° for 10 min. The hBD-2 and hBD3 ELISA development Kits (PeproTech, Rocky Hill, NJ) were then used according to the manufacturer's instructions. The detection limit of hBD2 ELISA was 8 pg/ml. The detection limit of hBD3 ELISA was 62 pg/ml.

### Data analysis

Each experiment was performed from at least three different donors, and within an experiment, each test condition was performed in duplicate. Values are shown as the Mean ± SD (standard deviation) or Mean ± SEM (standard error of the mean) from multiple experiments as indicated. Statistical significance was determined using one-way analysis of variance (ANOVA) among the groups followed by the two-tailed t-test. All statistical analysis was performed using JMP for Windows Release 6.0 (SAS Institute, NC). Differences were considered to be statistically significant at the level of p < 0.05.

## Results

### Human defensin expression in DCs *in vitro *and *in vivo*

**Figure 1:**
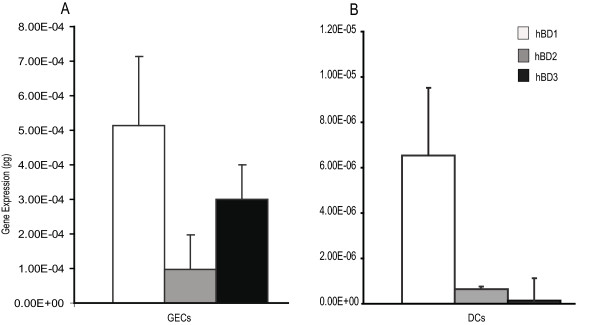
**(A, B). Basal level of defensin gene expression in GECs and iDCs in the absence of bacteria**. The absolute amount (picogram) of each defensin was determined using external plasmids standards in GECs (**A**) and iDCs (**B**). The expression of hBD1, hBD2 and hBD3 was normalized to housekeeping gene RPO. Results shown are the mean of three to five independent experiments. The error bars indicate SEM (standard error of mean).

To determine whether DCs express β-defensins, we compared the absolute gene expression levels in the absence of bacteria between unstimulated iDCs and GECs by quantitative real-time PCR using corresponding defensin plasmids as a standard curve. HBD1, hBD2 and hBD3 expression in GECs was significantly higher than that in iDCs (Figure 1A, B). HBD1, hBD2, and hBD3 expression of GECs is 80, 150, and 2000 fold higher, respectively, than that in iDCs. For both GECs and iDCs, the basal level of expression of hBD1 was highest among all the β-defensins. The expression level of hBD3 was much higher than that of hBD2 in GECs, while hBD2 expression was higher than that of hBD3 in DCs. Thus, both GECs and DCs express detectable levels of β-defensins, although the level is much lower in DCs.

### Differential response of defensins to oral bacteria between DCs and GECs

To compare the regulation of defensin expression between DCs and GECs, human iDCs and GECs were treated with graded doses of cell wall preparations from Gram-positive oral bacteria implicated in caries *A. naeslundii*, Gram-negative non-pathogen *F. nucleatum*, or Gram-negative periodontal pathogen *P. gingivalis*, and *E. coli *LPS. Each of these stimulants induced DC maturation on greater than 80% of cells as measured by expression of CD83 for mDCs [33].

**Figure 2:**
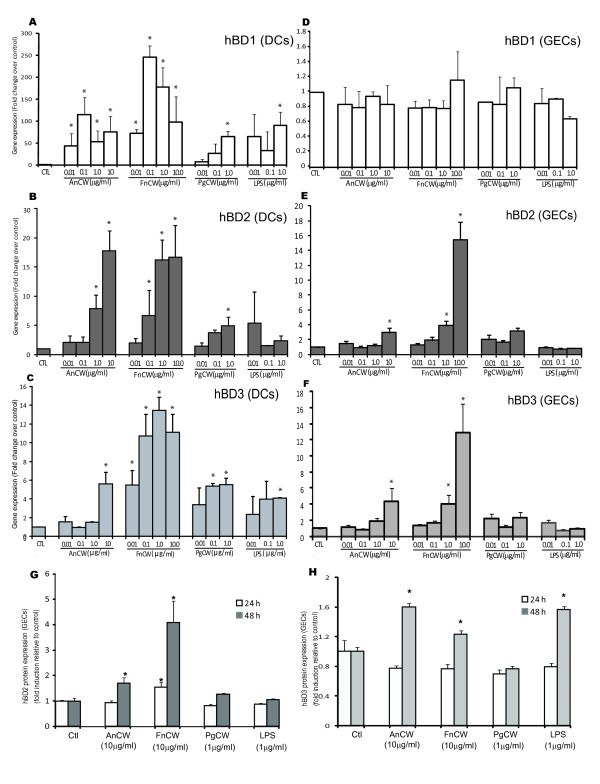
**Differential defensins expression in response to oral bacteria by DCs and GECs**. DCs (**A, B, C**) and GECs (**D, E, F**) were stimulated with graded doses of oral bacteria cell wall extracts (*A. naeslundii*, AnCW; *F. nucleatum*, FnCW; and *P. gingivalis*, PgCW) for 24 h. The gene expression of hBD1, 2, and 3 was quantified by quantitative real-time PCR. LPS was used as a positive control. **A, D**. hBD1 expression; **B, E**. hBD2 expression; **C. F**. hBD3 expression. Results are shown as mean fold change ± SEM over unstimulated controls. The data are average of three independent experiments performed in duplicate. GECs were stimulated with bacterial preparation for 24 and 48 h. Proteins were harvested, and hBD2 and hBD3 proteins in GECs were measured by ELISA (**G, H**). The concentration of defensins range was pg/ml. Inductions of hBD2 and hBD3 proteins are as fold induction relative to unstimulated controls. The data are average of two independent experiments performed in duplicate. Error bars represent the mean ± SEM. Asterisks indicate statistically significant difference compared to unstimulated control (Ctl) (*p < 0.05).

Gene expression of both hBD1 and hBD2 mRNA was induced in DCs by oral bacterial preparations (Figure 2A, B), but the level of induction varied with different bacteria. *A. naeslundii *and *F. nucleatum *stimulated greater hBD1 expression than *P. gingivalis *(2-10 fold higher) at the same dose (Figure 2A). Both *A. naeslundii *and *F. nucleatum *showed a bi-phasic hBD1 response with peak expression (120-220 fold) at 0.1 ug/ml cell wall preparation, but 60-fold induction at 1 μg/ml. In contrast, *P. gingivalis *showed dose-dependent induction with only 20-fold induction of hBD1 mRNA at 1 μg/ml. *A. naeslundii *and *F. nucleatum *gave a dose-dependent stimulation of hBD2 mRNA expression in DCs peaking at 16-18 fold over unstimulated controls, while *P. gingivalis *was a less effective inducer for hBD2 in DCs (Figure 2B). Controls with LPS showed approximate 100-fold stimulation of hBD1, but only minimal stimulation of hBD2 (< 5-fold). HBD3 gene expression in DCs followed a similar trend of hBD2 in response to bacterial preparation (Figure 2C). However, the hBD-3 PCR product gave two peaks in melting curve analysis in contrast to GECs. After eliminating the possibility of primer dimers and genomic DNA contamination, we assume these two peaks might be unreported (un-annotated) splice variants of the gene of interest in DCs. Further investigation will be performed in the future to clarify the difference between the hBD3 in DCs and GECs.

These results contrast with β-defensin expression in GECs in which hBD1was constitutively expressed and not altered by stimulation with bacteria, and hBD2 and hBD3 were induced in a dose-dependent manner by *F. nucleatum*, but unlike with DCs, not induced much by *A. naeslundii *and *P. gingivalis *(Figure 2D,E,F). LPS was not effective in upregulating hBD2 and hBD3 gene expression in epithelial cells at 24 h (Figure 2E, F).

Upregulation of hBD2 and hBD3 mRNA expression was accompanied by a similar increase in protein level of GECs as measured by ELISA (Figure 2G, H). GECs were stimulated with various bacterial preparations at 24 and 48 h. The time-dependent increases of hBD2 and hBD3 protein levels were observed after exposure to various bacterial preparations. HBD2 protein was significantly expressed in response to *F. nucleatum *(both 24 h and 48 h) and *A. naeslundii *(48h) in GECs at 48 h, which is consistent with the results obtained by mRNA gene expression at 24 h (Figure 2G). HBD3 protein was significantly expressed in response to *F. nucleatum*, *A. naeslundii *and LPS at 48 h, which is similar with trends observed by *F. nucleatum *and *A. naeslundii *at 24 h. HBD3 protein was significantly expressed in response to LPS at 48 h, which was not seen at 24 h (Figure 2H).

### DCs influence innate immune responses of GECs

**Figure 3:**
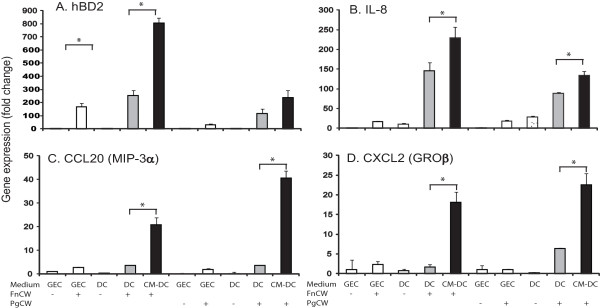
**Gene expression of hBD2 and other innate immune makers in GECs in response to conditioned media from bacterially stimulated DCs**. **A**. HBD2 mRNA upregulation in GECs in response to FnCW, and in the presence of conditioned medium (CM-DC, 1:2 dilution) from FnCW-stimulated DCs for 24 h. HBD2 mRNA was poorly expressed in GECs stimulated with PgCW (1 μg/ml), but expression was enhanced by the conditioned medium (CM-DC, 1:2 dilution) from PgCW-stimulated DCs. Controls include epithelial cells in GEC medium with or without added bacteria, and in DC medium with or without bacteria. Data are expressed as mean fold changes of experimental duplicates relative to unstimulated GECs after normalization to housekeeping gene RPO. Real-time PCR analysis of the additional markers for innate immunity (**B, C, D) **also show enhanced expression in GECs with conditioned medium from stimulated DCs (**B**: IL-8,**C**: CCL-20, **D**: CXCL2). Results are represented as mean ± SEM. *: p < 0.05 versus the relative control. Consistent results were obtained in two to three independent experiments for all markers.

In order to assess the influence of DCs on responses by GECs, we added conditioned medium from stimulated DCs to GEC cultures, and evaluated the expression of hBD2, IL-8, CCL20/MIP-3a, and CXCL2/GROβ by GECs in response to the cell wall preparations of *F. nucleatum *and *P. gingivalis*. Expression of each of these genes was markedly enhanced in the presence of DC-conditioned medium compared to DC medium from unstimulated cells as well as when compared to the regular GEC medium (Figure 3). Upregulation of these innate immune markers was 4- to 10-fold greater in the presence of DC-conditioned medium than simple stimulation of GECs. Conditioned medium from *F. nucleatum*-stimulated DCs was more effective at stimulation of hBD2 and IL-8 mRNA than medium from *P. gingivalis*-stimulated DCs, while medium from *P. gingivalis*-stimulated DCs yielded equal or greater stimulation of CCL20 and CXCL2 gene expression. These results suggest that stimulated DCs enhance innate immune responses in GECs via secretion of one or more bioactive effectors, and that the effect of the DCs on GECs is dependent on the specific bacterial stimulation.

**Figure 4:**
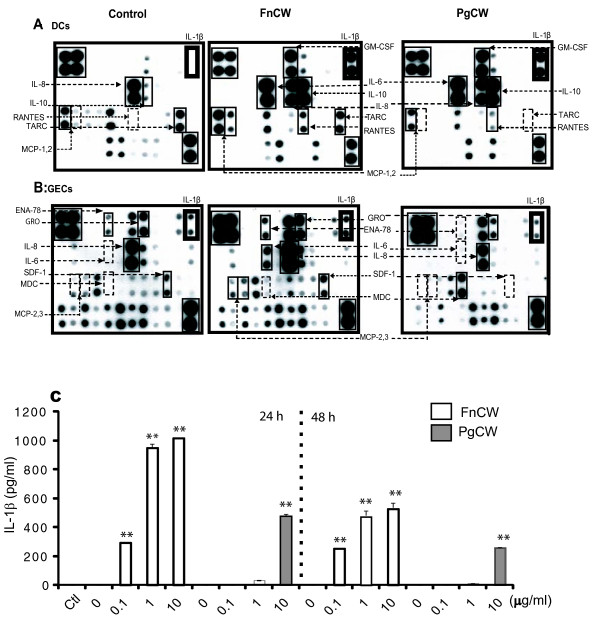
**Array analysis of the released cytokines and chemokines in response to *F. nucleatum *and *P. gingivalis *in DCs and GECs**. Each cytokine or chemokine is represented by duplicate spots. Supernatants (1 ml) of unstimulated cells or cells stimulated for 24 h with FnCW or PgCW were applied to RayBio human cytokine protein array III as described in Materials and Methods. The rectangles in the upper left and lower right portions of the arrays indicate positive controls. The upregulated cytokines and chemokines are indicated by solid rectangles; down-regulated or absent products are indicated by dashed rectangles. The cytokine array image represents one of two independent experiments (**A, B**). ENA-78, epithelial neutrophil-activating protein 78; GRO, growth regulated oncogene; MCP, macrophage colony stimulating factor; MDC, macrophage-derived chemoattractant; SDF-1, stromal cell-derived factor; TARC, thymus and activation-regulated chemokine. Note the upregulation of IL-1β in DCs with both bacterial preparations (bold rectangle). **C**. IL-1β in the cultured media from DCs after FnCW or PgCW stimulation. The cells were stimulated with FnCW or PgCW at the indicated dose and time points. The culture media were collected and IL-1β was quantified by ELISA. Results are represented as mean ± SD for two determinations in a representative experiment. Similar results were obtained with conditioned media from three different donors. (**: p < 0.001; *: p < 0.05). Ctl: medium only.

In order to identify the bioactive effectors that mediate the induction of hBD2 in GECs, and examine differential responses by oral bacteria between GECs and DCs, we initially evaluated cytokine/chemokine expression by protein array analysis of the culture media from both DCs and GECs after bacteria stimulation. *P. gingivalis *and *F. nucleatum *both triggered DCs to produce cytokines IL-1β IL-6 and IL-10 and GM-CSF as well as chemokine IL-8. However, the chemokines RANTES, MCP-1, 2, and TARC were upregulated more by *F. nucleatum *than *P. gingivalis *(Figure 4A). In contrast, the results for GEC stimulation, unlike with DCs, differed substantially for the two bacterial preparations (Figure 4B). *F. nucleatum*-treated GECs had increased expression of IL-6, IL-8, GRO, and MCP-3, while *P. gingivalis*-treated GECs increased MDC/CCL22 and reduced levels of ENA-78, SDF-1, and GRO compared to unstimulated control cells.

One of the most striking differences between DCs and GECs was IL-1β that was secreted by bacterially-stimulated DCs but not by stimulated GECs. Because IL-1β is a known inducer of hBD2, we quantified IL-1β by ELISA in culture media. IL-1β production increased in a dose-dependent manner in DC culture media after stimulation with either *F. nucleatum *or *P. gingivalis *(Figure 4C). *F. nucleatum *stimulation resulted in high levels of IL-1β in DCs even at as little as 0.1 μg/ml, and induced approximately 30-fold more IL-1β than *P. gingivalis *at the same dose. In contrast, IL-1β was not detected in the culture media of GECs stimulated with at the same dose of bacterial preparations (data not shown).

**Figure 5:**
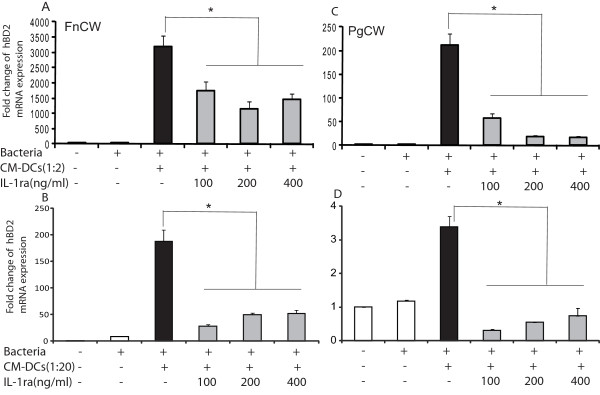
**Effects of IL-1ra on bacterially-induced hBD2 in cultured GECs**. Cells were stimulated by conditioned medium from DCs (CM-DCs) treated with FnCW (**A, B) **at 1:2 (**A**) or 1:20 dilution (**B**); or PgCW at 1:2 (**C**) and at 1:20 dilution **(D) **with or without IL-1ra (100, 200, 400 ng/ml). Expression of mRNA was analyzed by real-time PCR. Data are expressed as mean fold change of experimental duplicates compared to the expression of unstimulated cells for each condition after normalization to housekeeping gene RPO. Results are represented as mean of fold change ± SD for two determinations in a representative experiment. Similar results were obtained with cells from three different donors. Values are significantly different compared to the respective control (*: p < 0.05).

In order to test the functional role of IL-1β for hBD2 regulation, IL-1 receptor antagonist (IL-1ra) was added to the co-culture system. GECs were pretreated with graded doses of IL-1ra for 1 h, then stimulated with bacterial preparations in the presence of DC- conditioned medium (CM-DCs) with or without IL-1ra for 24 h. The IL-1ra doses used in this study did not affect cell viability. Recombinant IL-1ra partially blocked the expression of hBD2 mRNA induced by *F. nucleatum *in the presence of CM-DCs at 1:2 dilution (45-54%) (Figure 5A), and more completely blocked hBD2 expression when the condition medium was diluted tenfold (74-85%) (Figure 5B), compared with *F. nucleatum *stimulation alone. IL-1ra nearly completely blocked hBD2 expression in GECs stimulated with *P. gingivalis *in the presence of CM-DC at both dilutions (Figure 5C, D). IL-1ra also blocked CCL20, CXCL2 and IL-8 in *P. gingivalis*-stimulated cells in the same manner as hBD2, but was less effective in blocking CCL-20 and CXCL2 expression induced by *F. nucleatum*-stimulated GECs (data not shown). The fact that IL-1ra blocked several innate immune responses suggests that DC-derived IL-1β induces expression of these mRNA in GECs and that IL-1β can function in the dialogue or cross-talk between DCs and GECs.

### GECs influence innate immune responses of DCs via defensins

**Figure 6:**
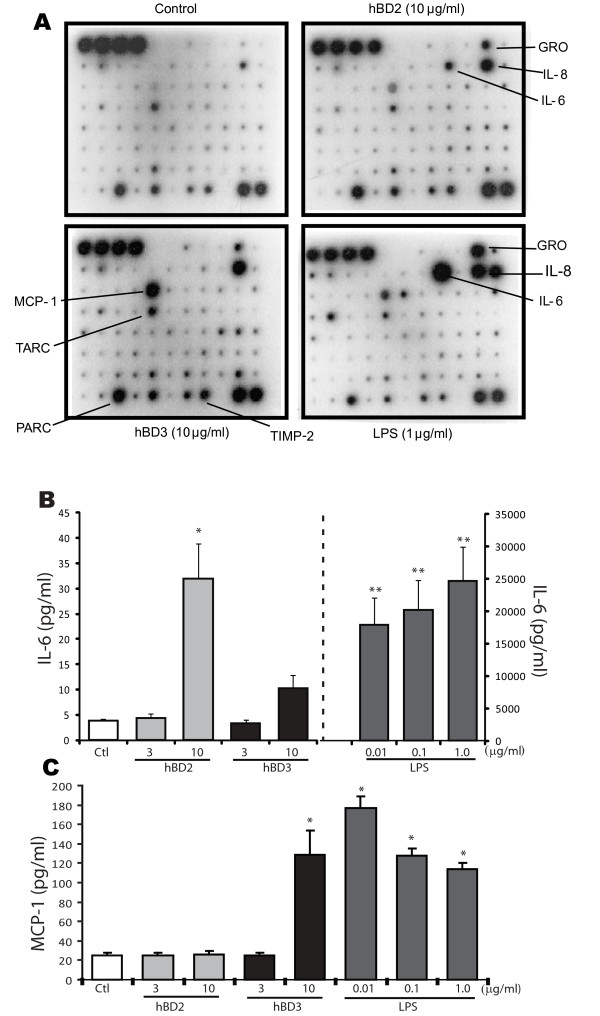
**A. Cytokine profiling in DCs stimulated with hBD2, hBD3 and LPS B, C. Assay of IL-6 and MCP-1 in culture media from DCs stimulated by β-defensins by ELISA**. **A: **The iDCs were treated with hBD2 (10 μg/ml), hBD3 (10 μg/ml), LPS (1 μg/ml) and untreated control for 24 h. Supernatants were analyzed by protein array as in Figure 4. The most differentially regulated cytokines are identified by arrow. **B, C: **The iDCs were stimulated with hBD2 (10 μg/ml) and hBD3 (10 μg/ml) at the indicated dose. The culture media were collected and IL-6 (**B**) and MCP-1 (**C**) were quantified by ELISA. Results are represented as mean ± SEM for three determinations from independent experiments. Note the difference in scale of **B **(right and left axis). HBD2 stimulated significantly greater IL-6 than hBD3, while hBD3 produced significantly greater MCP-1 than hBD2 (*: p < 0.05: **: p < 0.001).

To investigate how defensins from epithelial cells regulate DC responses, we evaluated the effects of hBD2 and hBD3 peptides on cytokines produced by DCs using semi-quantitative protein array analyses and ELISA. An overlapping but distinct profiling pattern of cytokines/chemokines was observed with treatment by hBD2 and hBD3 (Figure 6A). Both hBD2 and hBD3 treatment of iDCs resulted in increased secretion of GRO, IL-8, and MCP-1. In addition, hBD2 increases the secretion of IL-6, while hBD3 increased the secretion of TARC, PARC, and TIMP-2. LPS treatment resulted in significant increases in secretion of GRO, GRO-α, IL-6, IL-8, IL-10, and MCP-1.

The differential secretion of IL-6 and MCP-1/CCL2 by DCs treated with hBD2 and hBD3 was confirmed by ELISA (Figure 6B, C). HBD2 treatment significantly increased IL-6 secretion, although at much lower levels than LPS. In contrast, hBD3 treatment induced MCP-1 secretion in DCs to approximately the same extent as LPS.

**Figure 7:**
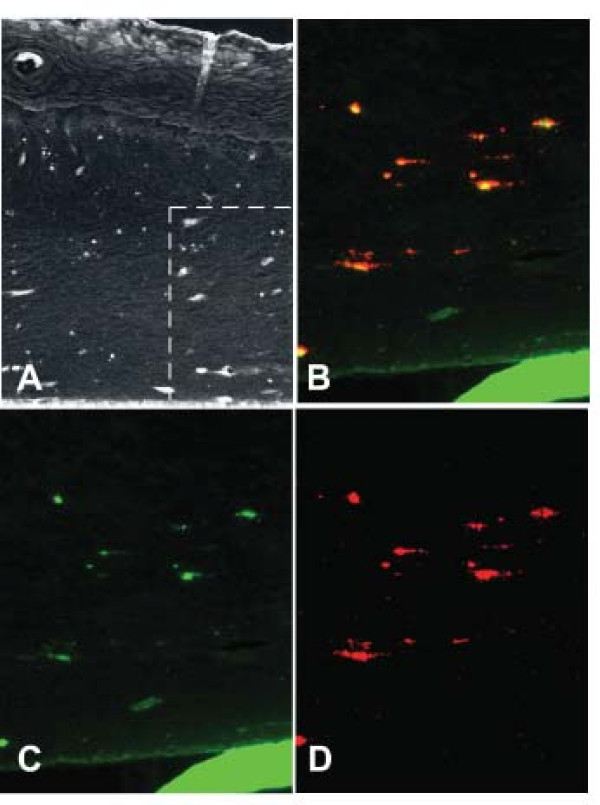
**(A-D). HBD2 associates with DCs in an oral tissue model**. HBD2 peptide was applied for 6 or 18 h to the surface of oral full thickness tissue model containing DCs. Immunohistochemical reaction of hBD2 polyclonal antibody (negative image) showing reaction in the upper layers of the epithelium and in a scattered distribution within the connective tissue. **B-D**. Double label immunofluroesence of an area similar to that indicated by the box in **A**, **B**. Double label; Anti-human HLA-DR monoclonal antibody with biotinylated anti-mouse IgG made in goat, streptavidin Alexafluor 594 (red) and anti-hBD2 polycloonal antibody made in rabbit with Alexafluor 488 labeled anti-rabbit IgG made in goat (green). **C**. HBD2 immunolocalization only (green). **D**. HLA-DR immunolocalization only (red). The solid green line visible in B and C is the supporting membrane for the tissue model. Controls without primary antibody were negative. Experimental control without added hBD2 showed no green fluorescence associated with HLA-DR positive cells. Original magnification: **A**: 20×, **B, D**: 60×.

In order to determine β-defensin interaction with DCs in a tissue model system, hBD2 was applied to the epithelial surface of a full-thickness mucosal tissue culture model system and incubated for 24 h. HBD2 adhered to DCs within the tissue model as detected by double label immunostaining (Figure 7).

## Discussion

Epithelial cells and DCs initiate and contribute to immune responses; however, the coordination of specific innate immune responses between these two cell types in response to various oral bacteria is not clear. In this report, we show that both epithelial cells and DCs express β-defensins and that products induced in response to oral bacteria by one cell type influence the characteristic responses of the other cell type. Apparently, the crosstalk so well defined between B and T cells [34] also can occur between GECs and DCs. Bacterially-activated DCs influence and significantly augment multiple innate immune responses of epithelial cells as shown by the expression of hBD2 as well as IL-8, CXCL2/GROβ and CCL-20/MIP3α in epithelial cells. This amplification is primarily mediated via IL-1β from DCs, since induction of these mRNAs in GECs was largely attenuated by IL-1ra. On the other hand, epithelial cells also influence the responses of DCs as shown by selective stimulation of cytokine/chemokine production by DCs in the presence of β-defensins. Furthermore, the responses of both cell types are dependent on the specific bacteria used for stimulation; this is demonstrated by the unique profiles of β-defensin expression as well as the differential cytokine/chemokine responses of both cell types when stimulated by cell wall preparations of various bacteria of different pathogenicity: *F. nucleatum*, *A. naeslundii *and *P. gingivalis*. Our findings highlight the differential and coordinated regulation of innate response in response to oral bacteria. These findings confirm previous studies and provide additional insights into how these two cell types interact in a network that may result in optimal immune regulation in response to various oral bacteria.

The β-defensins are mainly expressed by epithelial cells, although hBD1, but not hBD2, was previously detected in mature DCs, monocytes, and macrophages in response to LPS [35]. Our results show both hBD1 and hBD2 are expressed in DCs with basal level in iDCs approximately 100-fold lower than in epithelial cells. However, in contrast to epithelial cells, in which hBD1 is constitutively expressed and hBD2 and 3 are inducible, both hBD1 and hBD2 were inducible in DCs by bacterial exposure, while hBD3 was only weakly expressed. Furthermore, hBD2 diffusing from the epithelium associated with DCs as shown in an oral full thickness tissue model (Figure 7). Both hBD2 and hBD3 modulated expression of chemokines by DCs including IL-8 and GRO with differential dose-dependent upregulation of IL-6 by hBD2 and MCP-1 by hBD3. Our results agree and extend previous observations that β-defensins influence DC properties. Both hBD1 and hBD3 influence DC maturation with upregulation of costimulatory molecules [36,37], and hBD1 also stimulates expression of proinflammatory cytokines [37].

Oral bacteria differ in their effectiveness in inducing hBD1, hBD2 and hBD3 mRNA and proteins in GECs and DCs. The Gram-negative commensal, *F. nucleatum*, was more effective than a Gram-positive cariogenic bacterium, *A. naeslundii*, and both are more effective than the periopathogen *P. gingivalis *in upregulation of hBD1, 2 and 3 in DCs. This dose response pattern is similar to that shown by Chino et al [33] for chemokine and cytokine secretion by DCs. In addition, in DCs, hBD1 showed a bi-phasic response to the non-pathogens similar to the expression of MCP-1, while hBD2 expression was dose-dependent, similar to the IL-8 response [33]. Maturation of DCs was induced by direct exposure to oral bacteria [33] and by hBD3 [36] which is highly expressed by epithelial cells in response to *F. nucleatum *(Figure 2). Thus, low doses of commensal bacteria may play a role in immune surveillance by DCs under non-inflammatory conditions both by direct contact and by cross-talk from epithelial cells via β-defensins. On the other hand, epithelial cells also respond differently to different types of bacteria, as hBD2 and 3 gene expression is greatly induced by *F. nucleautm*, consistent with the immune-regulatory properties of this bacterium [31]. With greater exposure to commensal bacteria, or under inflammatory conditions, responses would be enhanced by secretion of IL-1β by DCs that stimulates epithelial cell expression of hBD-2 and CCL20, GRO and IL-8. The defensins further stimulate IL-6 and MCP-1 secretion by DCs, which might help to amplify the appropriate immune responses. Thus, epithelial cells and DCs may work together to express antimicrobial peptides and attract monocytes and neutrophils to fight infection in the gingival crevice, a critical factor for the health of the periodontium [27,38,39].

IL-1β, secreted by DCs, is the main cytokine responsible for mediating increased hBD2 expression in GECs, as previously demonstrated in epidermal keratinocytes and pulmonary epithelial cells [20,21]. IL-1ra also attenuated upregulation of other innate immune markers (CCL20, CXCL2) in parallel with that of hBD2. Thus, IL-1β can function in the dialogue or cross-talk between DCs and GECs. However additional factors, such as RANTES, which are differentially secreted by DCs treated with *F. nucleatum *vs. *P. gingivalis*, may also contribute to this cross-talk since IL-1ra was only partially effective in blocking *F. nucleatum *induced responses. Indeed, *P. gingivalis *(in the absence of its proteases) is a poor stimulant for hBD2 in GECs. However, the GECs can respond to conditioned medium from DCs stimulated with *P. gingivalis*, suggesting the importance of multiple cell types in the response *in situ*.

Epithelial secretion of defensins and chemokines that attract neutrophils is much reduced in the presence of the pathogen, *P. gingivalis*, consistent with its 'stealth-like' properties [40,41]. However, in response to this pathogen, epithelial cells secreted MDC/CCL22, an inflammatory chemokine that induces Th2 effector responses [42]. These trends could be related to events in gingival inflammation and periodontal disease. Jotwani and coworkers suggested that the prominent response in *P. gingivalis*-mediated periodontal disease is a Th2 effector response [43], supported by our findings. However, we did not find secretion of IL-8 or MCP-1 with our *P. gingivalis *cell wall stimulation in contrast to Kusumoto et al [44], which used a sonicated extract of *P. gingivalis*. Neutrophil chemotaxis is critical for periodontal health, and this process is interrupted in periodontal disease commonly associated with *P. gingivalis *[38,39]. IL-8 is expressed in *F. nucleatum*-stimulated GECs, and its expression is strongly enhanced by interaction with DCs stimulated with this commensal bacteria. However, overall levels are low in *P. gingivalis*-stimulated GECs and in the combination of *P. gingivalis*-stimulated DCs and GECs. Thus, both DCs and GECs distinguish between these bacteria and have specific responses.

The ability of DCs to stimulate immune responses is related to their activation and maturation status, and activated DCs are significant sources of chemokines that recruit other immune cells, including T cells, natural killer cells, monocytes and additional DCs [28]. The oral bacterial preparations used here induced the maturation of DCs, in agreement with our previous studies [33], as assessed by up-regulation of surface expression of CD83 (data not shown). Interestingly, both hBD2 and hBD3 induced the maturation of DCs as well, although to a lower extent (10%-30%) than that of bacteria preparations. Defensin treatment produced differential cytokine/chemokine profiles in DCs. Both hBD2 and hBD3 induced IL-8, GRO and MCP-1, in agreement with the findings in peripheral blood mononuclear cells [45]. However, DCs respond differently to hBD2 and hBD3; hBD2 induced IL-6, while hBD3 induced greater levels of MCP-1 than hBD2. The induction of IL-6 and IL-8 may be particularly important in attracting neutrophils and T helper 17 cells [46]. Multiple chemokines, including GRO, have microbicidal effects on both Gram-positive and Gram-negative bacteria [47]. MCP-1 acts as an attractant for monocytes and T-regs [48]. We also observed the increased secretion of TARC, PARC, and TIMP-2 in hBD3-treated DCs, which may attract more immune cells *in situ*. The evidence that hBD2 and 3 induced selective cytokine expression suggests that these defensins may play unique role in immune responses due to utilizing different receptors to stimulate DCs; TLR4, CCR6 and CD91 have been implicated as receptor for hBD2 [9,37,49] and TLR1 and TLR2 as receptors for hBD3 [36].

An intimate interaction between epithelial cells and DCs has been described in the gut to maintain immune homeostasis in response to various bacteria [50,51]. Our results for the first time demonstrate that a similar phenomenon of specific bacteria response coordinately may occur in the oral mucosa. Our findings show that DCs amplified the bacterially specific innate immune responses of GECs, while epithelial-derived defensins induced unique chemokine patterns, suggesting the existence of autoregulatory loop between DCs and GECs. GECs and DCs evoke characteristic cytokine patterns upon exposure to different bacterial stimuli and coordinately enhance each other's innate immune responses. Not only do defensins act as chemoattractants to immune cells, but we found that defensins also induce unique cytokine patterns, which could be crucial in amplifying immune responses to oral bacteria. These responses and cross-talk may result in discriminatory signals within oral tissue and gingiva in particular, and yield characteristic and appropriate immune responses in the state of health in the presence of non-pathogenic bacteria, and with inflammation in the presence of pathogens.

## Conclusions

The results suggest that cytokines, chemokines and β-defensins are involved in the interaction of DCs and GECs, and the responses are bacteria-specific. Differential and coordinated regulation between these two cell types may be important in the regulation of innate immune homeostasis and responses to pathogens in the oral cavity.

## Abbreviations

(hBD1, hBD2, hBD3): Human beta-defensin-1, 2, 3; (GECs): Gingival epithelial cells; (DCs): dendritic cells; (LCs): Langerhans cells; (FnCW): *Fusobacterium nucleatum *cell wall; (PgCW): *Porphyromonas gingivalis *cell wall; (AnCW): *Actinomyces naeslundii *cell wall.

## Authors' contributions

All authors have read and agreed with the contents of this manuscript. LY designed experiments, performed GECs culture, and the treatments of GECs and DCs, mRNA and proteins analysis, and drafted and revised the manuscript. TC conducted monocyte-derived DC, and detected DCs phenotypes. OVH conducted immunofluorescence. EAC provided DCs and participated in the discussion of manuscript. BAD conducted experimental design, coordinated and helped drafting of the manuscript. WOC contributed to discussion, drafting and revision of the manuscript.

## Acknowledgements

We thank Mr. Robert A. Underwood for assistance with fluorescence microscopy, Dr. Kevin Chen, Department of Microbiology and Immunology, for assistance with some co-culture experiments, and Ms. Janet Kimball for assistance with immunostaining. This work was supported by NIH/NIDCR grants R01 DE 013573, DE16381, AI52203 and P60 DE13061.
